# Evaluation methods to assess the efficacy of equinovarus foot surgery on the gait of post-stroke hemiplegic patients: A literature review

**DOI:** 10.3389/fneur.2022.1042667

**Published:** 2022-11-09

**Authors:** Nicolas de l'Escalopier, Cyril Voisard, Mona Michaud, Albane Moreau, Sylvain Jung, Brian Tervil, Nicolas Vayatis, Laurent Oudre, Damien Ricard

**Affiliations:** ^1^Université Paris Cité, Université Paris Saclay, ENS Paris Saclay, CNRS, SSA, INSERM, Centre Borelli, Paris, France; ^2^Service de Chirurgie Orthopédique, Traumatologie et Réparatrice des Membres, Service de Santé des Armées, HIA Percy, Clamart, France; ^3^Service de Neurologie, Service de Santé des Armées, HIA Percy, Clamart, France; ^4^SYSNAV, Vernon, France; ^5^Université Paris Saclay, Université Paris Cité, ENS Paris Saclay, CNRS, SSA, INSERM, Centre Borelli, Gif-sur-Yvette, France; ^6^Universitꃩ Sorbonne Paris Nord, L2TI, UR 3043, Villetaneuse, France; ^7^ENGIE Lab CRIGEN, Stains, France; ^8^Ecole du Val-de-Grãâce, Service de Santé des Armées, Paris, France

**Keywords:** equinovarus foot, hemiplegia, stroke, neuro-orthopedic surgery, gait analysis, IMU

## Abstract

**Introduction:**

The aim of this study was to realize a systematic review of the different ways, both clinical and instrumental, used to evaluate the effects of the surgical correction of an equinovarus foot (EVF) deformity in post-stroke patients.

**Methods:**

A systematic search of full-length articles published from 1965 to June 2021 was performed in PubMed, Embase, CINAHL, Cochrane, and CIRRIE. The identified studies were analyzed to determine and to evaluate the outcomes, the clinical criteria, and the ways used to analyze the impact of surgery on gait pattern, instrumental, or not.

**Results:**

A total of 33 studies were included. The lack of methodological quality of the studies and their heterogeneity did not allow for a valid meta-analysis. In all, 17 of the 33 studies involved exclusively stroke patients. Ten of the 33 studies (30%) evaluated only neurotomies, one study (3%) evaluated only tendon lengthening procedures, 19 studies (58%) evaluated tendon transfer procedures, and only two studies (6%) evaluated the combination of tendon and neurological procedures. Instrumental gait analysis was performed in only 11 studies (33%), and only six studies (18%) combined it with clinical and functional analyses. Clinical results show that surgical procedures are safe and effective. A wide variety of different scales have been used, most of which have already been validated in other indications.

**Discussion:**

Neuro-orthopedic surgery for post-stroke EVF is becoming better defined. However, the method of outcome assessment is not yet well established. The complexity in the evaluation of the gait of patients with EVF, and therefore the analysis of the effectiveness of the surgical management performed, requires the integration of a patient-centered functional dimension, and a reliable and reproducible quantified gait analysis, which is routinely usable clinically if possible.

## Introduction

The number of hemiplegic stroke survivors is constantly increasing (1). They classically develop spastic equinovarus foot (EVF), posing a challenge for rehabilitation (2, 3). The position in plantar flexion and inversion results from an imbalance in hindfoot forces due to muscular hypertonia associated with loss of effective motor control. The development of EVF is associated with muscle over-activity of the calf muscles, triceps surae, tibialis posterior, flexor hallucis longus, flexor digitorum longus (FDL), and brevis muscles, combined with paresis or weakness of the antagonist muscles, the tibialis anterior (TA), peroneus longus, and brevis. Over time, flexible deformities typically evolve into fixed deformities as a result of muscle shortening consequent to prolonged contracture (4). This raises serious problems with footwear, upright stance, transfer, and gait. For severe deformities, non-operative treatment is usually unsatisfactory; neuro-orthopedic surgery is recognized as effective in improving foot position in spastic EVF (5, 6), usually achieving improvement in functional scores [most notably the Goal Attainment Scale (GAS)] (7–11). Triceps spasticity, ankle range of motion, and gait velocity are improved. In addition, the need for walking aids is reduced (2, 6, 12–15). There are many surgical techniques, which act on the tendons, in order to lengthen them, to transfer them or both. The most frequently performed intervention is split anterior tibialis tendon transfer (SPLATT) (16). Most of the time we lengthen the triceps surae through the Achilles tendon lengthening (TL) or through the gastrocnemius and soleus aponeurectomy to treat equinus position (17). Other tendon transfers are the extensor hallucis longus transfer (18), anterior transfer of the FDL (19), split posterior tibial transfer (20), and peroneus brevis transfer (21). Another type of surgical procedure aims to act directly on the nerves to reduce the spasticity of certain muscles. These are called selective neurotomies (SN). In EVF, most of the time, they concern the branches of the tibial nerve and, by reducing its caliber, make it possible to reduce spasticity (22–24). More rarely, bony procedures (BPs) are necessary. The details of these different techniques and the current management strategy have been described in a more generic literature review on EVF of all etiologies (16), as well as following the DELPHI method (25). The choice of the techniques used and their possible combination is based on the clinical examination of the patient through a multidisciplinary approach (15, 16). If non-operative techniques are insufficient, neuro-orthopedic surgery can correct the equinus, reduce spasticity, and provide plantigrade support. However, it is essential to accurately analyze the gait disorder in a dynamic manner to propose a program adapted to the patient without risking aggravating his condition. The evaluation of the outcome of the surgery must also be based on a quantified and objective method of analysis and not only on a subjective clinical impression. Today, there is no standard, validated method for assessing gait improvement after post-stroke EVF surgery. Postoperative outcomes are heterogeneously assessed by quality of life scales, functional methods, such as the GAS, and by data from clinical examination, including splinting or barefoot walking. Change in gait pattern is difficult to analyze objectively and quantitatively, especially in longitudinal follow-up of patients. Quantified gait analysis techniques are effective but difficult to implement in routine clinical practice (26, 27). EVF is often evaluated by mixing different etiologies (TBI and cerebral palsy), and it seems important to us to evaluate a homogeneous patient population, here post-stroke EVF (28). The aim of this study was to realize a systematic review of the different ways, especially instrumental, used to evaluate the effects of surgical correction of post-stroke EVF deformities, including SN, tendon lengthening (TL), tendon transfer (possibly associated with TL), or BP, in post-stroke patients.

## Methods

In this review, we defined a stroke as “an acute neurological dysfunction of vascular origin with sudden (within seconds) or at least rapid (within hours) occurrence of symptoms and signs corresponding to the involvement of focal areas of the brain” (29). Details of the protocol for this systematic review were registered on PROSPERO and can be accessed at https://www.crd.york.ac.uk/prospero/display_record.php?ID=CRD42022300497.

### Search strategy and selection criteria

The literature search and analysis followed the Preferred Reporting Items for Systematic Reviews and Meta-Analyses (PRISMA) (30) and Meta-analysis of Observational Studies in Epidemiology (MOOSE) (31). We searched the MEDLINE (*via* PubMed), Cochrane Central, and Embase electronic databases to identify articles published before 1 June 2021 that measured the efficacy of neuro-orthopedic surgery on the gait of hemiplegic post-stroke hemiplegic patients. In addition, the gray literature was searched in Google Scholar, Opengrey.eu, Greylit.org, WorldCat, World Health Organization Clinical Trials Search Portal, ClinicalTrials.gov, and the European Union Clinical Trials Register. All reference lists and bibliographies of included studies were also reviewed for relevant articles. The following MeSH headings and keywords were used: “equinus, equinovarus, foot deformity, foot deformities, hemiplegia, hemiparesis, stroke, cerebrovascular disorders, orthopedics, neuro-orthopedic, neurorehabilitation, surgery, and gait analysis”.

### Selection criteria

As case series are probably the most frequent type of surgical report in the literature (32), it was decided not to restrict the selection to a specific study design. As a consequence, studies were included if they used either within-group pre-post treatment comparisons or between-group comparisons in a (at best randomized) controlled design. In addition, studies were required to meet the following inclusion criteria:

– investigating stroke in adults (irrespective of the phase of recovery);
– investigating the efficacy of surgical correction of EVF deformity (lengthening, release and/or transferring of muscles and/or tendons, and neurotomy);
– being written as a full-length article in the English, German, French, or Dutch language and being published in a peer-reviewed journal. If two or more articles were published by the same group, and if (within these articles) the etiology of the EVF deformity was comparable, only the study with the highest number of patients was included.

### Methodological quality assessment and data extraction

The Oxford CEBM levels of evidence were used to grade the selected studies (33). The methodological index for non-randomized studies (MINORS) was applied to further assess the quality of each study (34). Few validated instruments are available to assess the methodological quality of observational or non-randomized studies. MINORS is a validated list designed to assess the methodological quality of non-randomized (surgical) studies (either comparative or non-comparative) comprising 12 items, the last four items of which apply only to comparative studies. Items are scored as 0 (not reported), 1 (reported but inadequate), or 2 (reported and adequate). The maximum score is 16 for non-comparative studies and 24 for comparative studies. Because no (randomized) controlled trials were identified, no pooling of data was possible, neither in a meta-analysis nor in a best-evidence synthesis. We collected all the modes of evaluation of walking that were used, including the instrumental analysis of gait, the clinical analysis, the scores and scales used, and the subjective feelings of the patients. The GAS (35–37) was also reported if it was used.

## Results

### Study selection

**Figure 1:**
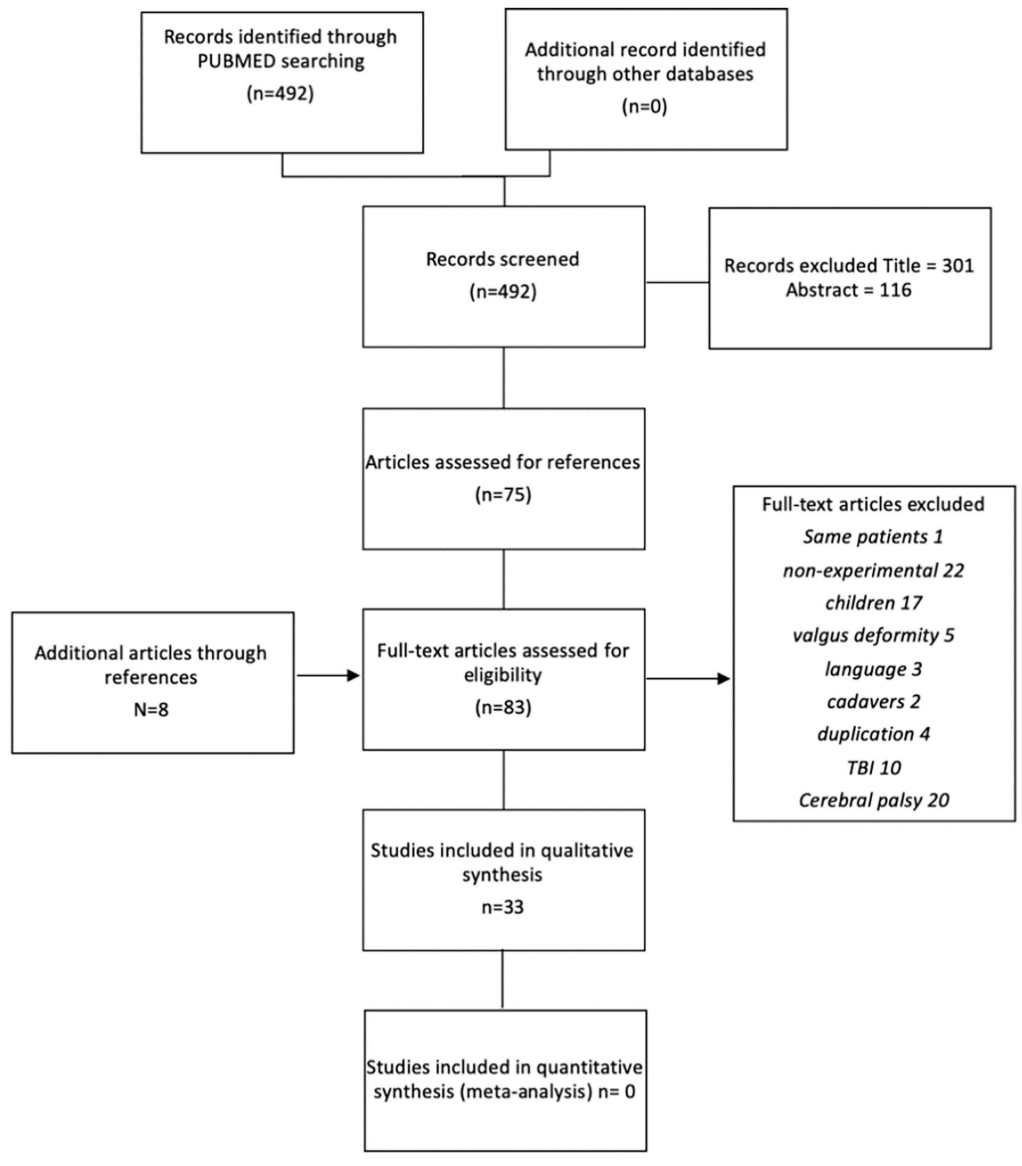
Study selection process.

Figure 1 shows the study selection process as a flowchart. The initial systematic search strategy in PubMed identified 492 relevant citations (available on request). The search in the other databases did not yield additional articles. On the basis of the title, 301 studies were excluded for the following reasons: non-surgical studies; botulinum toxin evaluation; pediatric populations; other pathologies, such as cerebral palsy or Traumatic Brain Injury (TBI); other neurological foot deformities; or non-neurological deformity. Another 116 studies were excluded based on their abstracts. Recurrent reasons for exclusion were the use of interventions that did not fit within our definition, such as reduction by external fixator or successive cast, and the use of patient populations with an etiology other than stroke. The full texts of the remaining 75 studies were examined. Screening the references of these studies revealed eight additional articles. From these 83 initially selected studies, 50 studies were excluded in the second instance.

### Methodological quality

The characteristics of the different articles included in the study are summarized in Table 1. The methodological quality of the studies and their heterogeneity did not allow for a valid meta-analysis. The median score was 11/16, which is low. Only 17 of 33 studies involved exclusively stroke patients.

**Table 1:** Characteristics of the different studies included.

**Authors**	**Study design**	**Minors**	**Number of subjects**	**Age**	**Time since injury**	**Type of intervention**	**Follow-up (months)**
Boffeli et al. (38)	Cases series, uncontrolled, prospective, single center, non-blinded	12	12 strokes	61 (54–73)	108 (11–240)	TL	29 (12–63)
Bollens et al. (39)	RCT, assessor blinded	20	8 strokes	50 (32–70)	30 (8–84)	SN	6
Buffenoir et al. (40)	Cases series, uncontrolled, prospective, multi center, non-blinded	12	34 strokes/55 patients	43.5 (12–54)	64 (3–320)	SN	10 (4–24)
Buffenoir et al. (41)	Historically controlled, prospective, monocenter, non-blinded	8	4 strokes/7 patients	41 (19–71)	37 (10–45)	SN	1
Buffenoir et al. (42)	Cases series, uncontrolled, prospective, mono center, non-blinded	11	9 strokes/15 patients	47 (22–66)	86 (12–84)	SN	15
Carda et al. (43)	Cases series, uncontrolled, retrospective, single center, non-blinded	13	177 strokes	50 (SD 14)	67	TT, TL	12
Decq et al. (44)	Cases series, uncontrolled, prospective, mono center, non-blinded	12	18 strokes/46 patients	36 (8–79)	96	SN	15 (8–28)
Delattre et al. (45)	Cases series, uncontrolled, retrospective, single center, no- blinded	6	9 strokes/10 patients	56 (30–80)	NC	TT, TL	51.7 (18–132)
Deltombe et al. (23, 46)	Cases series, uncontrolled, prospective, mono center, non-blinded	11	30 strokes	45 (20–69)	48 (15–218)	SN	24
Deltombe et al. (6)	Cases series, uncontrolled, prospective, mono center, non-blinded	12	18 strokes	55.7 ± 10.2	NC	SN, TT, TL	12
Edwards and Hsu (17)	Cases series, uncontrolled, retrospective, single center, non-blinded	9	9 strokes/11 patients	55 (23–72)	34	TT, TL	39 (12–79)
Gasse et al. (47)	Cases series, uncontrolled, retrospective, single center, non-blinded	6	14 strokes/22 patients	39.9 (17–76)	NC	TT, TL	6
Giannotti et al. (48)	Cases series, uncontrolled, retrospective, single center, non-blinded	8	47 strokes	56 ± 15	72 ± 60	TT, TL	1
Giannotti et al. (49)	Cases series, uncontrolled, prospective, mono center, non-blinded	11	24 strokes	55 (29–74)	60 (±36)	TT, TL	12
Keenan et al. (50)	Historically controlled, prospective, single- center, non-blinded	14	22 strokes /33 patients	40 (18–62)	NC	TT, TL	41 (17–53)
Khalil et al. (51)	Cases series, uncontrolled, retrospective, single-center, non-blinded	8	6 strokes/16 patients	38 ±15.2	112 ± 90	SN, TL	10.7 ± 6.8
Le Bocq et al. (52)	Cases controlled, prospective, single-center, non-blinded	11	23 strokes	57 (48–63)	28 (15–37)	SN	5
Lemos and Pereira (53)	Cases series, uncontrolled, retrospective, single-center, non-blinded	5	21 strokes/27 patients	49 (18–72)	7.1 (2–22)	TT, TL	29 (12–84)
Mazzoli et al. (54)	Cases series, uncontrolled, prospective, single-center, blinded	13	24 strokes	55 (29–74)	60 (36)	TT, TL	12
Mooney and Goodman (55)	Cases series, uncontrolled, retrospective, single-center, non-blinded	4	194 strokes	55 (17–84)	NC	TT, TL	6
Morita et al. (56)	Historically controlled, retrospective, single- center, non-blinded	11	125 strokes	57 (32–78)	23 (6–132)	TT, TL	33 (24–74)
Namdari et al. (57)	Cases series, uncontrolled, retrospective, single-center, non-blinded	12	64 strokes	54 (24–74)	65.7 (17–523)	TT, TL	12 (3–42)
Nonnekes et al. (58)	Cases series, uncontrolled, retrospective, single-center, no- blinded	6	10 strokes	48 (30–62)	84 (12–288)	BP, TL	7 (2–11)
Ono et al. (59)	Cases series, uncontrolled, retrospective, single-center, non-blinded	8	32 strokes /39 patients	(18–76)	>12	TT, TL	6
Pinzur et al. (26)	Cases controlled, prospective, single-center, non-blinded	13	36 strokes/54 patients	57 (17–77)	38 (12–204)	TT, TL	30 (24–62)
Reddy et al. (60)	Cases series, uncontrolled, retrospective, single-center, non-blinded	12	26 strokes	55 (23–72)	75	TT, TL	18 (6–48)
Rousseaux et al. (61)	Historically controlled, prospective, monocenter, non-blinded	14	34 strokes	50 (11–45)	45 (7–293)	SN	12
Rousseaux et al. (24)	Cases series, uncontrolled, prospective, monocenter, non-blinded	12	51 strokes	51 ±12	44 (11–304)	SN	24
Sindou and Mertens (62)	Cases series, uncontrolled, retrospective, single-center, non-blinded	9	19 strokes/53 patients	36 (6–68)	48 (2–17)	SN	36 (12–120)
Tracy (12)	Cases series, uncontrolled, retrospective, single-center, non-blinded	6	22 strokes/35 patients	40 (18–62)	36	TT, TL	32 (4–76)
Vogt (63)	Cases series, uncontrolled, retrospective, single-center, non-blinded	12	42 strokes/69 patients	47 (8–79)	50	TT, TL	44 (12–168)
Vogt et al. (64)	Cases series, uncontrolled, retrospective, single-center, non-blinded	6	80 strokes/132 (82 patients studied)	47 (11–78)	79 (13–486)	TT, TL	Mean 65
Yamamoto et al. (13)	Cases series, uncontrolled, retrospective, single-center, non-blinded	9	75 strokes	57	18	TT, TL	77

Type of intervention: SN, selective neurotomy; TL, tendon lengthening; TT, tendon transfer (possibly associated with TL) or BP, bone procedure.

### Surgical intervention

In view of the multitude of possible procedures, we preferred to classify the type of procedures performed into the following four categories: selective neurotomy (SN), tendon lengthening (TL), tendon transfer (possibly associated with TL), or bone procedure (BP). The studies and their associated categories are reported in Table 1. Ten studies (30%) evaluated only SNs, one study (3%) evaluated only TL procedures, 19 studies (58%) evaluated tendon transfer procedures, and only two studies (6%) evaluated the combination of tendon and neurological procedures, of which one evaluated only TL and one both TT and TL. Only one study (3%) evaluated exclusively BP procedures.

### Clinical assessment

The criteria used in the clinical evaluation of patients are reported in Table 2. There are a wide variety of scales, most of which have been validated in other indications, such as cerebral palsy (e.g., the Physicians Rating Scale) (65). Some seem relevant but are rarely used, such as the FPI-6 (72). Only three studies performed a GAS, one of which did not meet the Turner-Stokes criteria (11). The measurement of passive range of motion (ROM) was the most recurring criterion found, although its relevance for gait improvement was not assessed. On the contrary, the position of the foot during the oscillation phase, which is an essential element, was considered in only eight studies. Only 14 studies assessed patient satisfaction, mostly with simple numerical scales. Patient reported outcome measures were widely used, and only SF-36 (6) or SATISPART Stroke (39). GAS was used only one time (6), and a kind of unvalidated GAS one time (52).

**Table 2:** Clinical exams and type of gait analysis realized.

**Authors**	**Instrumental assessment of preoperative gait**	**Instrumental assessment of postoperative gait**	**Non-instrumental gait analysis**	**Clinical exam**	**Foot position stance**	**Foot position swing**	**Walking speed**	**Spatiotemporal parameters**	**Orthotic use**	**Walking capacity**	**Patient satisfaction**	**Gas**	**Others**
Boffeli et al. (38)	No	No	No	British foot score, MMST, ankle ROM	Yes	No	No	No	Yes	No	No	No	
Bollens et al. (39)	QGA	QGA	10MWT	Tardieu, MAS, MRC, PROM	No	No	Yes	Yes	No	No	SATISPART-stroke	No	SIAS
Buffenoir et al. (40)	No	No	10MWT	Equinus foot score, PROM, stretch reflex scale	No	No	Yes	Yes	Yes	Yes	No	No	
Buffenoir et al. (41)	Video-EMG gait analysis	Video-EMG gait analysis	No	General examination, PROM, tardieu, analytic exam	Yes	No	Yes	Yes	Yes	No	Patient satisfaction score/10	No	
Buffenoir et al. (42)	No	No	No	Ashworth, stretch reflex, equinus foot score, PROM	Yes	No	No	No	Yes	Yes physicians rating scale (37), independent walking score	Yes	No	Electrophysiological and biomechanical with specified devices
Carda et al. (43)	QGA	QGA	Spatiotemporal parameters	Walking handicap score	No	No	Yes	Yes	No	No	No	No	
Decq et al. (44)	No	No	Kinematic gait assessment on video, individual qualitative assessment	Ashworth, tardieu, PROM	Yes	Yes	No	No	Yes	No	No	No	
Delattre et al. (45)	No	No	Video record of gait	FPI-6 (65)	Yes	No	No	No	No	No	Yes	Yes	
Deltombe et al. (23, 46)	No	No	10MWT, video record of gait	Ashworth, MRC, PROM	Yes	Yes	Yes	Yes	No	No	No	No	
Deltombe et al. (6)	No	No	10MWT, video record of gait	Modified Ashworth Scale (63), Tardieu, MRC, PROM/AROM	Yes	No	Yes	Yes	Yes	Yes, FWC (66), ABILOCO (67)	Yes	Yes	SIAS, SF-36
Edwards and Hsu (17)	No	No	No	Kling et al. classification (68)	No	No	No	No	No	No	No	No	
Gasse et al. (47)	No	No	No	No	No	No	No	No	No	No	Yes, %	No	
Giannotti et al. (48)	QGA	QGA	No	No	Yes	Yes	Yes	Yes	No	No	No	No	
Giannotti et al. (49)	QGA	QGA	No	No	No	No	Yes	Yes	No	No	No	No	
Keenan et al. (50)	QGA	QGA but only 20 patients	Yes, descriptive	Ambulatory scale	Yes	Yes	No	No	Yes	No	No	No	Dynamic EMG
Khalil et al. (51)	COP	COP	No	No	No	No	No	No	No	No	No	No	
Le Bocq et al. (52)	QGA	QGA	No	MAS, MRC, PROM, LL-FAS, NFAC (69)	Yes	Yes	Yes	Yes	Yes	Yes	Yes	Yes (-4.4)	
Lemos and Pereira (53)	No	No	No	Perception of improvement on gait	No	No	No	No	Yes	Yes	Yes	No	Questionnaire non- valid
Mazzoli et al. (54)	No	No	6MWT	NPRS, CGI-C, FAC, RMI, WHS	No	No	Yes	Yes	Yes	Yes	No	No	
Mooney and Goodman (55)	No	No	No	Quantitative assessment of walk	Yes	No	No	No	No	Yes	No	No	
Morita et al. (56)	No	QGA 25/125	No	Walking ability	Yes	No	No	No	Yes	Yes, walking ability	No	No	
Namdari et al. (57)	Yes	No	No	Viosca score (qualitative assessment of gait)	Yes	Yes	Yes	Yes	Yes	Viosca (70)	No	No	
Nonnekes et al. (58)	QGA	QGA	No	FAC	No	No	Yes	Yes	No	FAC, 1-10	No	No	
Ono et al. (59)	No	No	No	Qualitative gait analysis	Yes	No	No	No	No	No	Yes	No	
Pinzur et al. (26)	QGA	QGA	No	PROM	Yes	Yes	No	Yes	Yes	Non	Yes	No	
Reddy et al. (60)	QGA	No	No	Qualitative gait analysis	No	No	No	No	Yes	Viosca (70)	No	No	
Rousseaux et al. (61)	No	No	10MWT	MAS, MRC, FAC, RMA (71)	No	No	Yes	Yes	Yes	FAC	Yes, 0-3	No	
Rousseaux et al. (24)	No	No	Gait pattern, 10MWT	MAS, PROM, ICF, MRC, RMA, FAC	Yes	No	Yes	Yes	Yes	FAC	Yes, 0-3	No	
Sindou and Mertens (62)	No	No	No	Ashworth, PROM, AROM, MRC	Yes	No	No	No	Yes	No	No	No	
Tracy (12)	No	No	No	Qualitative gait analysis	Yes	Yes	No	No	Yes	Yes	No	No	
Vogt (63)	No	No	No	Descriptive clinical exam	Yes	No	No	No	Yes	Yes, ability to walk 1–5	Yes	No	
Vogt et al. (64)	No	No	No	Qualitative gait analysis focused on knee flexion	Yes	Yes	No	No	Yes	Yes, functional autonomy 1–5	No	No	
Yamamoto et al. (13)	No	No	No	Qualitative gait analysis	No	No	Yes	No	Yes	Yes	Yes	No	

MMST, Manual muscle strength testing; A-P/ROM, Active/Passive range of motion; MAS, Motor assessment scale; MRC, Medical research council; SIAS, Social interaction anxiety scale; FPI-6, Foot posture index; 10MWT, 10 meter walk test; 6MWT, 6 minute walk test; FWC, Functional walking categories; QGA, Quantitative gait analysis; EMG, Electromyogram; LL-FAS, Lower limb function assessment scale; NFAC, New functional ambulation classification; RMI, Rivermead mobility index; NPRS, Numeric pain rating scale; CGI-C, Clinical global impression of change; WHS, Walking handicap score; RMA, Rivermead motor assessment.

### Gait assessment

Instrumental gait analysis refers to all modes of gait analysis using objective and quantified parameters. The types of instrumental analyses that were performed in the included studies are presented in Table 2. Quantitative gait analysis (QGA) is the gold standard for the study of human gait using reflectors attached to the body. It consists of video (kinematic and kinetic recording using digital cameras), an optoelectronic system, and a force platform. BP refers to a simple baropodometric and COP displacement study, and some studies performed only an elementary analysis including speed and number of steps. BP refers to a simple baropodometric and COP displacement study, and some studies performed only an elementary analysis including speed and number of steps.

Eleven studies conducted a non-instrumental walking analysis. Six studies used the 10-Meter Walk Test (10MWT), and one study used the 6-Minute Walk Test (6MWT). Note that the 6MWT did not show any correlation with the functional scores achieved in this study (54). In one study, only the walking velocity was evaluated over 10 m (56). Four studies used sensorless video recording, allowing for secondary measurement of analytical joint mobility by several observers (6, 23, 44, 45). Instrumental gait analysis was performed in only 10 studies (26, 39, 41, 43, 48–52, 58) (Table 3), and only six studies associated it with clinical and functional analyses (Table 2). It was only performed pre-operatively in one study and postoperatively in one study. In two studies, it was not performed on all the patients who were operated on. Regarding spatiotemporal parameters, the most frequently used were elementary parameters that did not require any specific indicators, such as speed or step length.

**Table 3:** Characteristics and methodology of instrumental gait analysis performed to assess postoperative hemiplegic gait.

**Study**	**Protocol**	**St Parameters**	**Kinematic**	**Kinetic**
Bollens et al. (39)	Three-dimensional analysis (38, 40). Segmental kinematics were recorded by eight infrared cameras (200 Hz) with the Eliclinic system (BTS, Milan, Italy), while the patients were walking at a comfortable speed on a treadmill (Mercury LTMed, HPCosmos, Nussdorf, Germany). The ground reaction forces (GRFs) were synchronously recorded (200 Hz) using four strain gauges located under the corners of the treadmill (Pharos System Inc, Rochester, NY), and the net joint movements in the sagittal plane were computed from the GRF, kinematic, and anthropometric data.	–	Ankle and knee ROM	–
Buffenoir et al. (41)	Video analysis with self-adhesive markers that were placed on the lateral surface of the spastic limb at fixed points. These markers were used to measure the range of knee and ankle flexion during computerized analysis of the video recording.	10MWT	Ankle and knee ROM	–
Carda et al. (43)	S.A.F.Lo. protocol (17, 46). Nine 15 mm reflective markers (lower prominence of the sacrum, posterior superior iliac spines, lateral femoral condyles, lateral malleoli, and fifth metatarsal heads). ELITE [ELaborazioneImmaginiTElevisive] three-dimensional system (BTS SpA, Milan, Italy) with polyelectromyography and two piezo-electric force platforms (Kistler AG, Winterthur, Switzerland). All patients were assessed while barefoot.	Self-selected speed, swing velocity, cadence, step length, stride length, and step Body speed of advancement during healthy swing phase (mean linear velocity of the marker placed on the sacrum during the swing phase of the unaffected limb. The measure is representative of body progression over the paretic foot).	Maximum ankle dorsiflexion	Center of pressure (COP) posterior-anterior progression (47, 55) COP posterior-anterior regression COP posterior-anterior crossover Posterior-anterior GRF positive and negative peak Vertical GRF Ankle power absorption and generation peak during stance phase
Giannotti et al. (48, 49)	Six camera motion capture system, (sMart-dX, BTS Bioengineering, Milan, Italy) and two force platforms (Kistler aG, Winterthur, Switzerland) with markers placed according to the conventional protocol (57)	Gait symmetry and stability: anterior step length and double support time Balance: step width Walking ability: speed, cadence, and stride length	Ankle DF at initial contact, maxi- mum DF at stance and maximum DF at swing	–
Keenan et al. (50)	Bidirectional slow-motion video recording with GRF	Walking velocity Cadence Stride time	–	–
Le Bocq et al. (52)	Two-dimensional video recording system. The spatiotemporal gait parameters were evaluated using an 8-meter GAITRite^®^ mat (CIR Systems Inc., Sparta, NJ, USA). Two trials (each over a total distance of 10 m [i.e., starting about a meter before the mat and finishing about a meter afterwards]) were performed at a comfortable speed and then averaged. These two objective gait measurements were performed barefoot and assistive.	Gait speed and cadence Non-paretic and paretic step lengths **Gait asymmetry** (as defined by Patterson et al.: non-paretic step length/paretic step length) (73): Paretic swing Total stance Single support phase durations (as a percentage of the gait cycle)	Gait Assessment and Intervention Tool (GAIT) (59) Ankle and knee ROM	-
Nonnekes et al. (58)	Reflective markers were placed at anatomical landmarks according to the full-body Plug-in-Gait model (61). Marker positions were recorded by an eight camera 3D motion analysis system (Vicon Motion Systems, United Kingdom) at a sample rate of 100 Hz. GRFs under both feet were recorded at a sample rate of 1000 Hz by two force plates (AMTI Custom 6 axis composite force platform, USA). Kinetics and kinematics were calculated with **Vicon Clinical Manager software. Kinematic**	Walking speed Cadence Stride length, step length and single- support time of both the paretic and nonparetic leg. **Step length asymmetry** was quantified by using a step length ratio defined as the difference in step length between the paretic and nonparetic side divided by the average step length of the paretic and nonparetic side (positive values indicate a larger paretic step compared to the nonparetic step).	Ankle ROM	Internal peak ankle moment Peak ankle power of the paretic and nonparetic leg.
Pinzur et al. (26)	Preoperative dynamic EMG and electrogoniometry	Double support phase length Stance phase length	-	-
Khalil et al. (51)	Data were collected using the F-Scan in-shoe system. It allows people to walk in normal shoes, using an insole measuring device to detect changes in **COP displacements or plantar pressures**. The recording frequency is 50 Hz, and the data are recorded and processed in the system's software (F-Scan Mobile Research 5.72 software).	–	–	Anteroposterior displacement of the COP measured from the most anterior to the most posterior points Lateral deviation of the COP measured from the two most lateral points Posterior Margin of foot contact measured from the most posterior point of heel contact to the most poste- rior point of the COP trajectory

Three studies evaluated symmetry by various means. Le Bocq et al. (52) used non-paretic step length divided by paretic step length as defined by Patterson et al. (73); for Nonnekes et al. (26), step length asymmetry was quantified by using a step length ratio defined as the difference in step length between the paretic and non-paretic sides divided by the average step length of the paretic and non-paretic sides (positive values indicate a larger paretic step compared to the non-paretic step). For Giannotti et al. (48, 49), gait stability and symmetry were represented by anterior step length and double support time but without any analysis. The authors did not recover the raw data (51, 58), and data analysis was performed by engineers or software, so the method used to obtain the results was not explicit and therefore not reproducible. Kinematics analysis evaluated ankle ROM and sometimes knee ROM. Ankle dynamic ROM was the most frequently used criterion. The only criteria used in the analysis were closed-chain joint kinematics and spatiotemporal parameters. No scores or other assessment methods were used. To the best of our knowledge, inertial measurement systems, largely used in other context of walk assessment in other conditions (74), have never been studied in this type of study.

### Outcomes

Functional results according to the main criterion used by the studies, as well as complications, and follow-up in months are summarized in Table 4. None of the studies had unfavorable results, and the complication rate was quite low. However, the wide variety of procedures and the differences in the collection of complications or residual deformities did not yield global conclusions. The response to the primary endpoint of the studies is reported in the right-hand column. There was no homogeneity in the evaluation criteria.

**Table 4:** Primary endpoint results of the different studies.

**Authors**	**Strokes/subjects**	**Complications**	**Residual or recurrent deformities**	**Follow-up (months)**	**Main criterion**
Boffeli et al. (38)	12	0	25%	29 (12–63)	BFS 55-35, *p* = 0.0022
Bollens et al. (39)	8	3	NC	6	Ankle stiffness L-path significantly decreased from T0 (482.95 ± 163.20 N m rad−1) to T1 (172.17 ± 102.88 N m rad−1)
Buffenoir et al. (40)	34/55	9%	0	10 (4–24)	Equinus foot score decreased from 1.54 preoperative to 0.273 after neurotomy (t test; p < 0.0001)
Buffenoir et al. (41)	4/7	0	0	1	Mean patient satisfaction score 7.7/10
Buffenoir et al. (42)	9/15	0	1	15	90% improvement of clinical spasticity scores, 20% improvement of walking scores
Carda et al. (43)	177	8	3	12	WHS 3.78 (SD 1.31) to 5.13 (SD 1.04) *p* < 10^−3^
Decq et al. (44)	18/46	0	0	15 (8–28)	100% improvement of equinus deformity
Delattre et al. (45)	9/10	0	3	51.7 (18–132)	−5.9 to −3.5 FPI-6
Deltombe et al. (23, 46)	25/30	0	0	24	Significant decrease in triceps surae spasticity, an increase in gait speed, and a reduction in equinus and varus in swing and stance phases at 2 months postoperatively.
Deltombe et al. (6)	18	8	0	12	GAS score [median (quartile 1-quartile 3)] observed at T1 [52.3 (46.6–59.1)] and T2 [52.3 (46.6–66.0)] *p* < 0.05
Edwards and Hsu (17)	9 / 11	1	3	39 (12–79)	King et al. three Good, four Excellent, two Poor
Gasse et al. (47)	14 / 22	2	2	6	90% contracts fulfilled; 100% were satisfied (41%) or very satisfied (59%) with their operation
Giannotti et al. (48)	47	0	0	1	Ankle df increased 1 month after surgery at all investigated gait phases (Wilcoxon test, *p* < 0.0001)
Giannotti et al. (49)	24	0	0	12	Variables relating to ankle kinematics improved toward their normal values at 1 month after surgery.
Keenan (50)	22/33	0	0	41 (17–53)	100% correction of deformity; improvement of ambulatory status.
Khalil et al. (51)	6/16	NC	NC	10.7 ± 6.8	COP variation for the paretic limb, a significant increase of AP was observed after block (13.5 vs. 12.3 cm, *p* = 0.02) and after surgery (13.7 vs. 12.3 cm, *p* = 0.03). A significant decrease of PM was observed after surgery (4.5 vs. 3.3 cm, *p* < 0.001) with no more difference between two limbs (2.8 vs. 3.3 cm, *p* = 0.44).
Le Bocq et al. (52)	23	3	2	5	TNN had a very marked effect on the level of spasticity and the range of motion in dorsiflexion (*p* < 10^−3^).
Lemos and Pereira (53)	27	11	0	29 (12–84)	Patients experienced frank improvement in terms of gait, orthostatic posture, self-esteem and quality of life.
Mazzoli et al. (54)	24	0	0	12	All variables but the 6MWT were significantly improved (Wilcoxon test, *p* < 0.05) at T1 or T2 and this remained until the 12-month mark.
Mooney et al. (55)	194	9	6	6	Improvement of subjective walking capacity
Morita et al. (56)	125	1	46	33 (24–74)	91/125 able to walk without a brace
Namdari et al. (57)	64	NC	NC	12 (3–42)	All patients were corrected to a plantigrade foot at final follow-up examination; 49 of 64 patients (76.6%) had an improved ambulatory status postoperatively as measured by Viosca score
Nonnekes et al. (58)	10	0	NC	7 (2–11)	Walking speed significantly improved by 32% after surgery (0.38 ± 0.20 m/s to 0.50 ± 0.17 m/s, *p* = 0.007).
Ono et al. (59)	39	No data	Toe curling	6	In all cases, correction of the equinovarus deformity was achieved and maintained.
Pinzur et al. (26)	36/54	2	2	30 (24–62)	Equinus deformity was corrected in all patients and 59% of them were brace-free.
Reddy et al. (60)	26	0	NC	18 (6–48)	Reduction in the use of nonoperative therapies in caring for patients with this condition.
Rousseaux et al. (61)	34	13	0	12	TNN (M3, M6 and Y1) resulted in a more significant effect than BTI (D15, M2 and M5) on most of the measures: ankle plantar flexor spasticity, range of movement in dorsiflexion and eversion, foot position in upright situation, functional ambulation categories (barefoot), RMA, gait velocity (comfortable condition), subjective benefit and use of walking aids.
Rousseaux et al. (24)	51	10	0	24	Neurotomy definitely reduced spasticity and improved motor control on antagonist muscles while improving balance, walk, and the RMA.
Sindou and Mertens (62)	19/53	6	11	36(12–120)	Complete suppression of disabling spasticity
Tracy (12)	22/13	1	6	32 (4–76)	91% removal orthosis
Vogt (63)	42/69	12	11	44 (12–168)	Significant improvement in patient autonomy (*p* < 0.001), demonstrated by an improved ability to ambulate independently and a decreased need to wear orthopedic shoes (*p* < 0.001) and orthoses (*p* < 0.001), as well as an increased ability to wear normal shoes (*p* < 0.001).
Vogt et al. (64)	80/132 *(82 studied)*	6	8	Mean 65	80/82 patients were able to walk barefoot, 74 reported an increase in their walking distance, and 73 could regularly wear normal shoes.
Yamamoto et al. (13)	75	NC	14	77	Correction was maintained in 74% of patients; 79% did not use an orthosis; 51% could bathe unassisted; and 76% were satisfied with the results.

BFS, Bristol Foot Score; WHS, Walking Handicap Score; FPI-6, Foot Posture Index; GAS, Goal Attainment Scale; COP, Center of Pressure; TNN, Tibial Nerve Neurotomy; 6MWT, 6 Minute Walk Test; RMA, Rivermead Motor Assessment.

## Discussion

To the best of our knowledge, our review is the only one dealing exclusively with the management of EVF in adult hemiplegic post-stroke patients, focusing on evaluation methods of the impact of the surgery on gait. Indeed, in our opinion, to better specify the management and the consequence of possible surgical procedure in the gait pattern, it is necessary to study cohorts of patients with the same pathology. Some studies mixed patients with amyotrophic lateral sclerosis, multiple sclerosis, Little's disease, TBI, and stroke (42, 44). Moreover, it is the first review to include both the classical clinical analysis and the instrumental analysis of walking, which should become essential in the years to come. It highlights not only the lack of consensus on the clinical criteria used in the evaluation of gait but also the poor access to quantified analysis in routine clinical practice. The main limit of this review lies in the fact that no meta-analysis was possible because of the statistical weaknesses of the included studies. This has already been noticed in previous reviews on the subject (28).

### Surgical strategy

There is great heterogeneity of attitudes, divided between neurological and tendinous procedures, due to the fact that they are performed by different surgical teams. For several years, the performance of all these procedures by the same teams has made it possible to refine the indications and to perform combined procedures (6, 15, 16, 25). Few series have analyzed the results of this global management. It is important that future series do not take into account only one procedure or another, but the “neuro-orthopedic” management itself.

### Clinical assessment

Numerous scales and measurement methods have been used, but none of them has imposed itself. This clearly shows the absence of a validated generic scale for evaluating walking in hemiplegics. On the contrary, the measurement of joint kinematics in analysis, although easily achievable and reproducible, does not give a good idea of what really happens in the closed chain during walking. Moreover, the type and importance of the global deficit are variable, and the resulting functional discomfort is specific to each patient. For this reason, it is extremely difficult to obtain a functional scale that can be adapted to each situation. Some have simply used a scale of satisfaction and the feelings of the patients with different subjective criteria (53).

The GAS is a method for quantifying progress on personal goals. Turner-Stokes's guide to the GAS is a method for quantifying progress toward personal goals (11). Turner-Stokes's guide and the use of Kiresuk's T-score (75) are the most widely used GAS-based approaches in rehabilitation (37). This personalized analysis allows to directly evaluate the success of the intervention, according to the defined objective. It represents a sensitive and specific analysis of the result. Indeed, in other pathologies, the GAS and the overall clinical impression have shown a significant correlation (7, 9). Moreover, in rehabilitation, the GAS is more sensitive to change than the Barthel Index and the Functional Independence Measure (8–10). In some studies, the GAS was the only scale capable of detecting change after treatment (8, 76). Standard scales sometimes show no change, while the GAS goal is achieved. The main reason for this is that the goals and fixed GAS often do not correspond to any of the items in the standard scales (77). For us, this functional analysis must now be part of the systematic clinical analysis of the outcome of the intervention. The limitation of this method lies in the definition of pre-operative goals, since it must be sufficiently ambitious but still achievable. Regarding the evaluation of spasticity, as explained by Deltombe et al. (22), although the Ashworth scale is commonly used in the literature, it is confounded by contracture, as increased resistance to movement is not only exclusively dependent on stretch reflex activity but also due to increased stiffness as a result of contracture. The Tardieu scale seems more appropriate, especially to evaluate triceps spasticity (78). Reddy et al. (60) used the evaluation of the reduction of non-operative care associated with an EVF deformity, which seems to be a relevant criterion (60).

### Gait assessment

Traditional non-instrumental scales have moderate effectiveness. The 6MWT does not appear to be a relevant indicator. As used in the study by Mazzoli et al. (54), it shows no difference pre- and postoperatively, while all functional scores are improved. Its use is, therefore, not relevant in this indication. The 10MWT provides some information, notably on step length and speed. However, this analysis does not detail the intrinsic quality of walking. As we said before, there is no correlation between the analysis of the open and closed chain gaits, and if the analytical analysis is easily done and traceable in the medical record, global analysis of gait is more difficult to assess in an objective way. In some retrospective studies, data concerning the exact position of the foot during the gait cycle were too unequally reproduced in patient files to be properly exploited (64). This pre- and postoperative comparison of the gait analysis seems essential to evaluate the effect of the procedure. For example, the comparison of pre- and postoperative joint kinematics is a reliable and reproducible criterion that can be measured by instrumental analysis (43). For this purpose, instrumental gait analysis methods are of considerable help. The gold standard is QGA, which provides more precise data for assessing surgical outcome, to improve the surgical program in spastic EVF and define more standardized strategies (27). While QGA represents the gold standard, the availability of facilities and immediacy of results makes QGA challenging to use in routine clinical. Indeed, there is no consensus on QGA indices, as such a consensus requires a team of engineers and physicians trained in interpreting such data. There is also a delay between acquisition and final analysis. Finally, the analysis can only be conducted in dedicated premises, often located far from where the patient lives or is being followed. For all these reasons, QGA is difficult for doctors to implement in routine clinical practice and postoperative follow-up. Presently, there are some simple tests for assessing dynamic balance in consultation, basically consisting of observing the patient walking and quantifying gait on an equipped walkway. However, we saw that instrumental gait analyses were scarce and of widely varying quality to evaluate EVF treatment in post-stroke adults. In addition, no validated and reproducible indicators were used. For example, only three studies evaluated pitch symmetry, and the three indicators were different. Most studies used instrumental analysis only to collect simple spatiotemporal data or joint kinematics data, which represents a limited contribution. No team collected the raw signal data for analysis, and they used the parameters provided by the brand's software but not their own algorithm. Moreover, the raw data were not accessible in open source. Moreover, QGA provides precise data on locomotion, but they require large and specific spaces, are very expensive (between €10,000 and €40,000), and hardly suited for everyday medical practice. Recently, a study by Mazzoli et al. (54) showed a good correlation between indices based on ground reaction force and clinical and functional variables. Since the acquisition of ground reaction forces does not require patient preparation, it can be used in clinical routine and especially for postoperative evaluation (79). An alternative is to use combined accelerometric and gyroscopic data on an inertial measurement unit (IMU). IMUs have the advantage of being lightweight, inexpensive, and easy to use in practice. It has been validated for clinical use in gait assessment in patients with osteoarthritis or neurological pathology, such as post-stroke hemiplegia (74, 80–83). Another advantage is the possibility to perform ambulatory measurements over a longer period of time in the patient's environment (84), which is not feasible with QGA. On the contrary, the comparison with the norms of healthy subjects is a criterion that is not often used but seems to be correlated with walking improvement. This cross-sectional step study represents a complementary element in the evaluation of postoperative improvement (26).

## Conclusion

Neuro-orthopedic surgery for post-stroke EVF is becoming better defined. However, outcome assessments are not yet well established. The complexity of the evaluation of gait of patients with EVF, and therefore the analysis of the effectiveness of the surgical management performed, requires the integration of a patient-centered functional dimension, as well as a reliable and reproducible quantified gait analysis, and if possible usable in routine clinical practice. Therefore, it seems necessary, in future, to compare the results of a systematic instrumental analysis with the functional results.

## Data availability statement

The original contributions presented in the study are included in the article/supplementary material, further inquiries can be directed to the corresponding author.

## Author contributions

Nl'E: writing and submission of the article. MM, CV, SJ, and BT: writing and correction. AM, NV, LO, and DR: conception and correction. All authors contributed to the article and approved the submitted version.

## Conflict of interest

Author MM was employed by company SYSNAV. Author SJ was employed by company ENGIE. The remaining authors declare that the research was conducted in the absence of any commercial or financial relationships that could be construed as a potential conflict of interest.

## Publisher's note

All claims expressed in this article are solely those of the authors and do not necessarily represent those of their affiliated organizations, or those of the publisher, the editors and the reviewers. Any product that may be evaluated in this article, or claim that may be made by its manufacturer, is not guaranteed or endorsed by the publisher.
